# Longitudinal Dynamics of Skin Bacterial Communities in the Context of Staphylococcus aureus Decolonization

**DOI:** 10.1128/spectrum.02672-21

**Published:** 2022-04-06

**Authors:** Stephanie A. Fritz, Todd N. Wylie, Haley Gula, Patrick G. Hogan, Mary G. Boyle, Carol E. Muenks, Melanie L. Sullivan, Carey-Ann D. Burnham, Kristine M. Wylie

**Affiliations:** a Department of Pediatrics, Washington University School of Medicine, St. Louis, Missouri, USA; b McDonnell Genome Institute, Washington University School of Medicine, St. Louis, Missouri, USA; c Department of Molecular Microbiology, Washington University School of Medicine, St. Louis, Missouri, USA; d Department of Pathology & Immunology, Washington University School of Medicine, St. Louis, Missouri, USA; e Department of Medicine, Washington University School of Medicine, St. Louis, Missouri, USA; Wayne State University

**Keywords:** *Staphylococcus aureus*, microbiome, skin, decolonization, households

## Abstract

Decolonization with topical antimicrobials is frequently prescribed in health care and community settings to prevent Staphylococcus aureus infection. However, effects on commensal skin microbial communities remains largely unexplored. Within a household affected by recurrent methicillin-resistant S. aureus skin and soft tissue infections (SSTI), skin swabs were collected from the anterior nares, axillae, and inguinal folds of 14 participants at 1- to 3-month intervals over 24 months. Four household members experienced SSTI during the first 12-months (observational period) and were prescribed a 5-day decolonization regimen with intranasal mupirocin and bleach water baths at the 12-month study visit. We sequenced the 16S rRNA gene V1–V2 region and compared bacterial community characteristics between the pre- and post-intervention periods and between younger and older subjects. The median Shannon diversity index was stable during the 12-month observational period at all three body sites. Bacterial community characteristics (diversity, stability, and taxonomic composition) varied with age. Among all household members, not exclusively among the four performing decolonization, diversity was unstable throughout the year post-intervention. In the month after decolonization, bacterial communities were changed. Although communities largely returned to their baseline states, relative abundance of some taxa remained changed throughout the year following decolonization (e.g., more abundant *Bacillus*; less abundant *Cutibacterium*). This 5-day decolonization regimen caused disruption of skin bacteria, and effects differed in younger and older subjects. Some effects were observed throughout the year post-intervention, which emphasizes the need for better understanding of the collateral effects of decolonization for S. aureus eradication.

**IMPORTANCE** Decolonization with topical antimicrobials is frequently prescribed to prevent Staphylococcus aureus infection, but the effects on commensal skin bacteria are undetermined. We found that decolonization with mupirocin and bleach water baths leads to sustained disruption of bacterial communities.

## INTRODUCTION

Staphylococcus aureus is a pathobiont (commensal bacterium with pathogenic potential [1]) causing conditions ranging from asymptomatic colonization to cutaneous abscesses (often recurrent) to invasive, life-threatening infection (2, 3). The emergence of community-associated methicillin-resistant S. aureus (CA-MRSA) strains over the past 2 decades has posed a significant burden in health care and community settings (4, 5). As MRSA colonization is an endogenous source for infection, preventive measures commonly include decolonization, the application of topical antimicrobials (e.g., mupirocin) or antiseptic agents (e.g., chlorhexidine or dilute bleach water), to eradicate S. aureus carriage (6).

Across multiple study populations, the practice of topical antimicrobial application has been demonstrated to reduce MRSA colonization burden and infection incidence. Hence, decolonization has become a common practice in health care and community settings (4–11). However, few studies have investigated the effect of topical antimicrobials on endogenous bacterial communities (12–14). Topical antimicrobials may disrupt the skin microbiota, accommodating the presence and prosperity of potential pathogens, analogous to dysbiosis of the intestinal microbiota with systemic antibiotics (15). As decolonization initiatives for infection prevention continue to expand (8, 16), it is essential that we understand the capacity of these topical agents to disrupt commensal bacterial communities and associated consequences.

The objective of the present study was to assess bacterial community characteristics and evaluate disruption of bacterial communities in a large household of 14 individuals that participated in a 24-month, longitudinal study of CA-MRSA, some of whom were prescribed a decolonization regimen (17–21).

## RESULTS

### Cohort characteristics.

Samples were collected from the nares, axillae, and inguinal folds from the index patient and 13 household contacts up to 10 times over 24 months. Seven participants were male and seven were female, with a median age of 9 years (range 0–37). Thirteen participants were colonized with S. aureus at least once during the 24-month study. The median number of time points at which individuals were colonized with S. aureus was 5: 5 participants were colonized with MRSA, 2 with methicillin-susceptible S. aureus (MSSA), and 6 with both MRSA and MSSA (either simultaneously at different body sites or at different sampling time points). Overall, 8 S. aureus strain types (by repPCR) were recovered from the 14 household members over the 24 month longitudinal study. The index patient was persistently colonized with the same MRSA strain type from the 3 month sampling through the 18 month sampling. Some of the household contacts were also intermittently colonized with the index patient’s MRSA strain type, while others were intermittently colonized with different strain types (both MRSA and MSSA).

### Bacterial community characteristics in the pre-intervention and post-intervention study periods.

**FIG 1:**
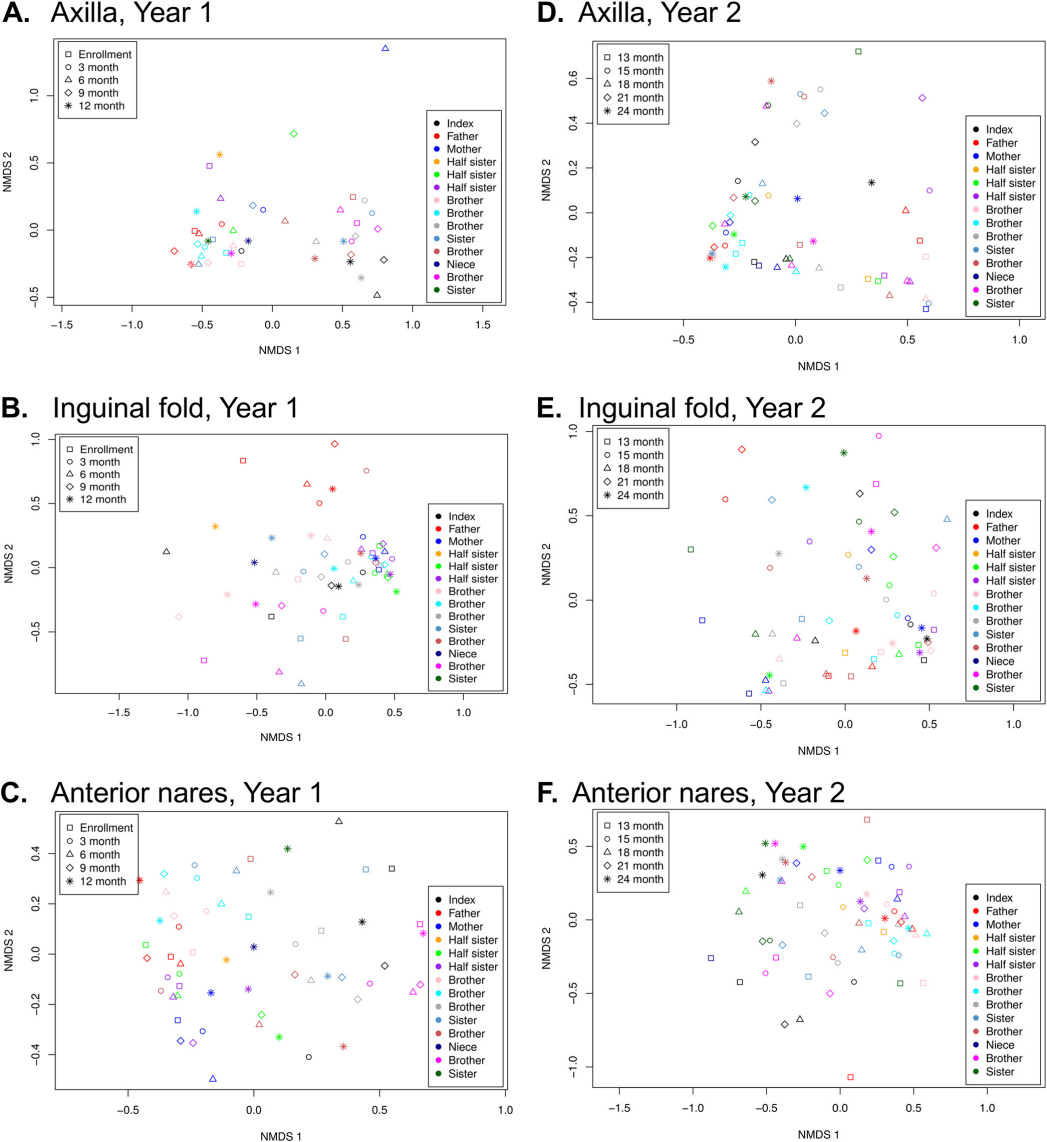
Ordination analysis. NMDS ordination plots are shown for samples collected from the (A) axillae during year 1 (the observational period), (B) inguinal folds during year 1, (C) anterior nares during year 1, (D) axillae during year 2 (the post-intervention period), (E) inguinal folds during year 2, and (F) anterior nares during year 2. Each sampling time point is indicated by a shape (key in the top left corner of each figure), and each individual in the study is indicated by a color (key in the bottom right corner). Clustering by subject was significant by Adonis testing, *P* < 0.001, for panels A, B, C, and F.

We analyzed the bacterial community characteristics longitudinally and determined effects of the decolonization intervention. We first compared the bacterial community structures in each sample using nonmetric multidimensional scaling (NMDS). The axilla, inguinal fold, and nares samples collected throughout the observational period clustered by subject (*P* < 0.001) but not by time point (Fig. 1A to C). At the 12-month study visit, the index patient and 3 household contacts who had developed SSTIs during the 12-month observational period were assigned the decolonization intervention. During the post-intervention period, the axillae and inguinal fold samples no longer significantly clustered by subject (Fig. 1D and E, *P* > 0.05), though the nares samples continued to cluster by subject (Fig. 1F, *P* < 0.001).

**FIG 2:**
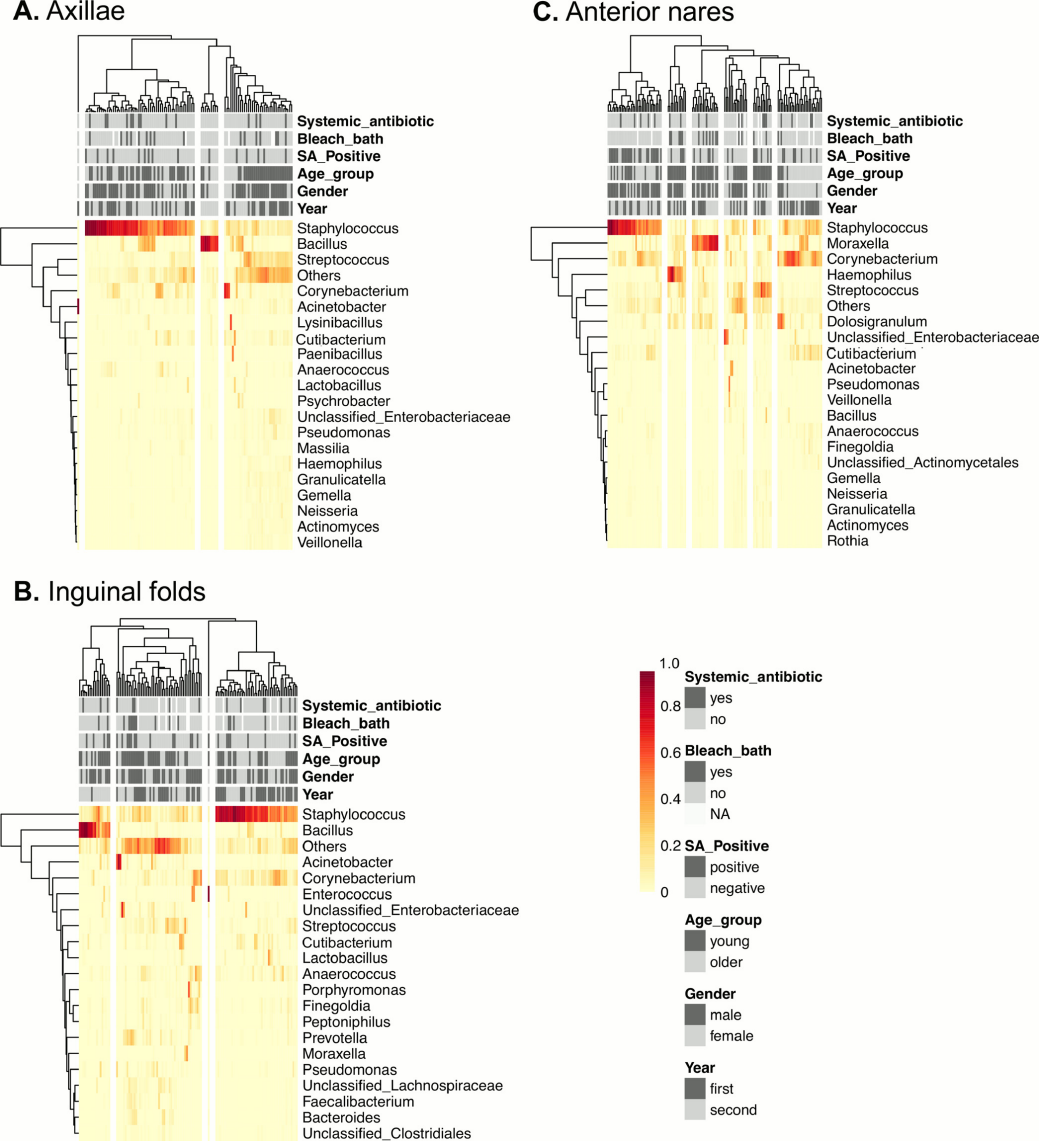
Bacterial communities and associations with participant characteristics and study procedures. The relative abundance of the top 20 taxa for the (A) axillae, (B) inguinal folds, and (C) anterior nares are shown on the heatmaps. Each row represents a different taxon, and each column represents a different sample. Low to high abundances are represented on the gradient from yellow to red. Clinical features are shown in gray and black at the top of each heatmap, and the key is at the bottom right side of the figure.

We generated a clustered heatmap of bacterial communities to visualize associations between participant characteristics, study procedures, and bacterial community structures (e.g., Staphylococcus-dominant communities). In the axillae (Fig. 2A), younger age associated with the cluster of samples containing moderate Staphylococcus, Streptococcus, and “Other” bacteria (a group representing lower abundance organisms) (Fig. 2A, right cluster). One cluster, defined by a high level of *Bacillus*, contained only samples collected during the post-intervention period (Fig. 2A, middle cluster). In the inguinal folds (Fig. 2B), there was a cluster dominated by *Bacillus* predominantly comprised of samples collected during the post-intervention period (Fig. 2B, left cluster). In the nares (Fig. 2C), samples collected from participants who had taken additional bleach baths (beyond what was prescribed for the intervention) did not cluster with samples dominated by Staphylococcus (Fig. 2C, left cluster). The same Staphylococcus-dominant cluster was associated with samples that were S. aureus positive by culture. The younger age group (<12 years old at the beginning of the study) was associated with several clusters, including those with high Haemophilus, *Moraxella*, or Streptococcus (Fig. 2C, middle clusters). Samples from females in the older age group associated in a cluster with moderate levels of Staphylococcus and *Corynebacterium* (Fig. 2C, right cluster). The study period of sample collection (observational period versus post-intervention period) did not associate with specific clusters (Fig. 2C).

**FIG 3:**
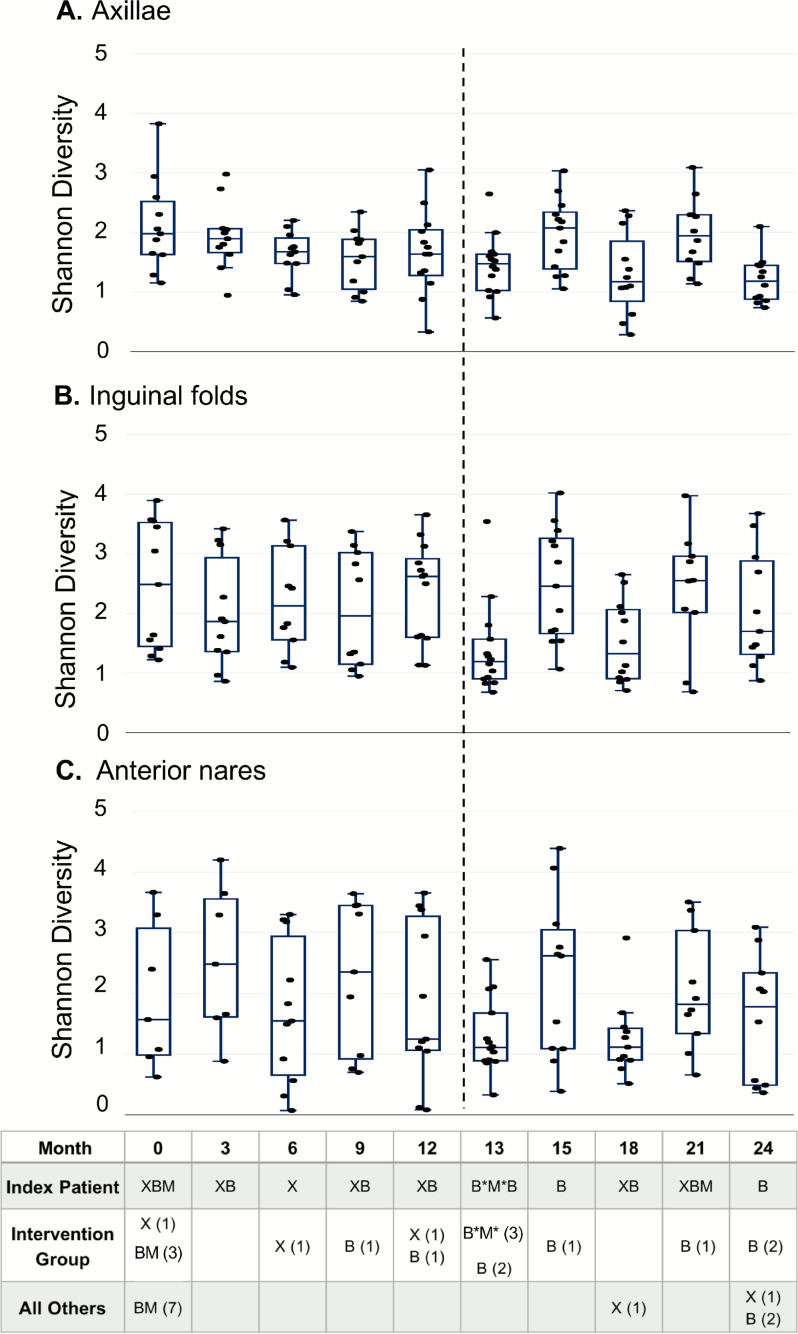
Shannon diversity over time. The Shannon diversities of the samples from the (A) axillae, (B) inguinal folds, and (C) anterior nares are shown at each sampling time point. Pre-intervention and post-intervention time points are divided by the vertical dashed line. The data are displayed with box and whisker plots, which include the median (solid horizontal line), interquartile ranges (box), and min and max (whiskers), excluding outliers. The table at the bottom of the figure indicates interval skin infections and bleach baths and mupirocin applications performed during each interval between study visits: X represents interval skin infection; B*M* indicates the 5-day trial intervention of bleach baths and twice daily mupirocin applications; B represents bleach baths performed outside those prescribed for the trial; M represents mupirocin applications performed outside those prescribed for the trial; (#) indicates the number of individuals in the group affected by SSTI or performing topical antimicrobials or antiseptics during the interval.

We evaluated the alpha (within sample) diversity over time. During the observational period, the median Shannon diversity in the axillae, inguinal folds, and nares was stable, even in the context of sporadic bleach baths performed by some household members (Fig. 3), as were other measures of alpha diversity (not shown). 1 month after the prescribed decolonization intervention (performed by the index patient and 3 other household contacts), the Shannon diversity of samples from all household members trended lower in all body sites (Fig. 3, 13 months). In the post-intervention period, diversity was unstable in the axillae, inguinal folds, and nares (Fig. 3), oscillating between lower and higher values across samplings. Using linear mixed-effects models, we determined that that the Shannon diversities of the communities were significantly associated with the sampling visit, largely reflecting dynamic changes in diversity associated with the decolonization intervention, and the age of the subjects sampled, with higher diversity communities recovered from younger subjects (axillae: visit *P* = 0.01, age *P* = 0.009; inguinal folds: visit *P* = 0.0006, age *P* = 0.003; nares: visit *P* = 0.003, age *P* = 0.04). S. aureus colonization or the development of SSTI in the interval prior to or after sampling was not associated with Shannon diversity, although the numbers of samples in these comparative groups was small.

### Bacterial community characteristics associated with age.

**FIG 4:**
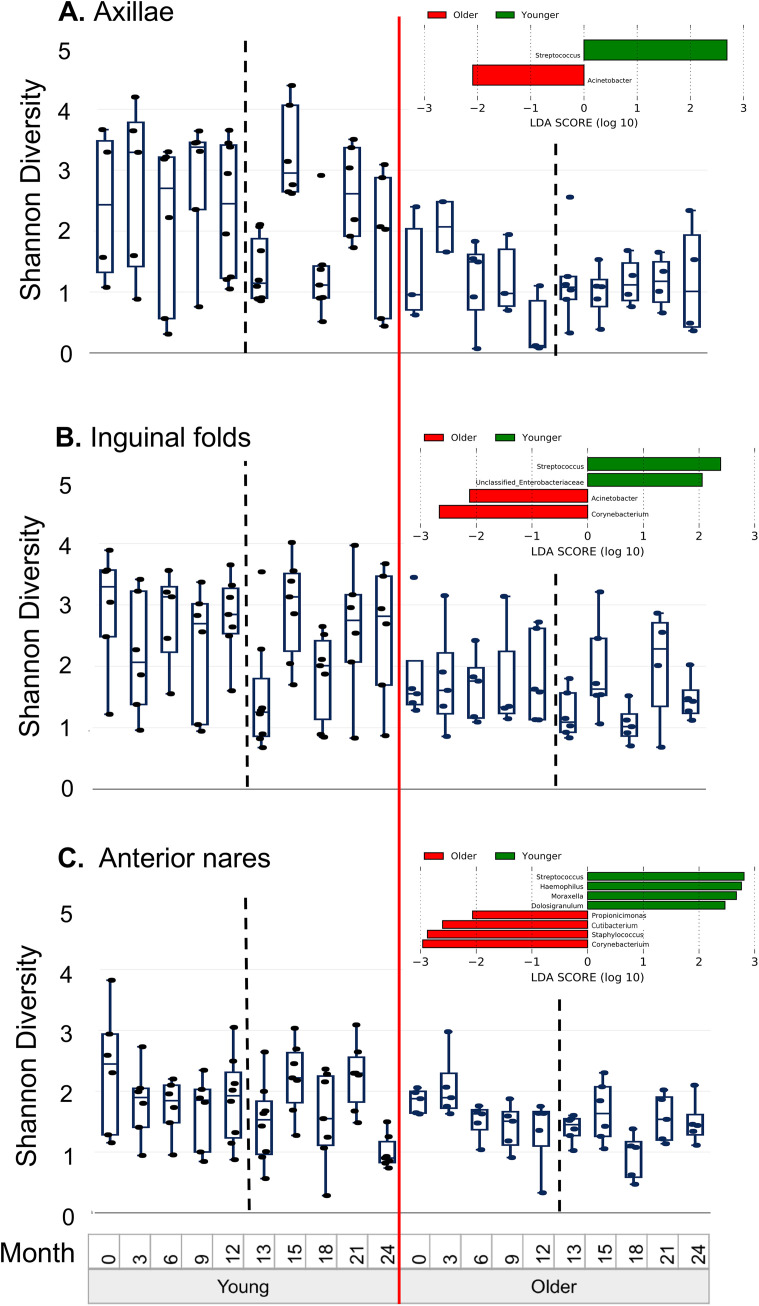
Shannon diversity over time stratified by age. The Shannon diversities of the samples from the (A) axillae, (B) inguinal folds, and (C) anterior nares are shown at each sampling time point, with the subjects <12 years of age at the beginning of the study on the left and the subjects ≥12 years on the right, divided by the vertical solid red line. Pre-intervention and post-intervention time points in both age groups are divided by vertical dashed black lines. The data are displayed with box and whisker plots, which include the median (solid horizontal line), interquartile ranges (box), and min and max (whiskers), excluding outliers. Taxa that distinguish the older and younger subjects for each body site (identified by linear discriminant analysis [LefSe]) are inset into each panel, with red bars showing taxa more prevalent in older subjects and green bars showing taxa more prevalent in younger subjects.

Since age was a driving factor in community structure and diversity metrics, we compared samples from subjects who were <12 years old at the beginning of the study with those ≥12 years old. The Shannon diversity of the communities at each body site was higher in younger participants than older participants (Fig. 4A to C). In the younger subjects, greater dynamic change was observed in samples collected during the post-intervention period compared to older subjects, and Shannon diversity in the axillae was strongly associated with the sampling visit (linear mixed-effects model, *P* = 0.0005); no such association was observed in older subjects (*P* = 0.2). In inguinal fold samples, Shannon diversity was associated with the visit in younger subjects (*P* = 0.014) and older subjects (*P* = 0.002). In the nares, younger subjects showed variability in Shannon diversity over time, although this was not statistically significant (*P* = 0.07). In older subjects, the variability in the Shannon diversity of nares samples was associated with sampling visit (*P* = 0.0001).

We then evaluated specific taxa that distinguished samples from the subjects who were <12 years old compared with those who were ≥12 years old. In the axillae, Streptococcus spp. were associated with the younger subjects, while Acinetobacter was associated with older subjects (Fig. 4A). In the inguinal folds, the groups were distinguished by Streptococcus spp. and unclassified *Enterobacteriaceae* in the younger subjects and Acinetobacter and *Corynebacterium* in the older subjects (Fig. 4B). The nares from the younger subjects had higher levels of Streptococcus, Haemophilus, *Moraxella*, and *Dolosigranulum*, while the older subjects had more *Propionicimonas*, *Cutibacterium*, Staphylococcus, and *Corynebacterium* (Fig. 4C).

### Effects of decolonization on bacterial community structure and taxa.

**FIG 5:**
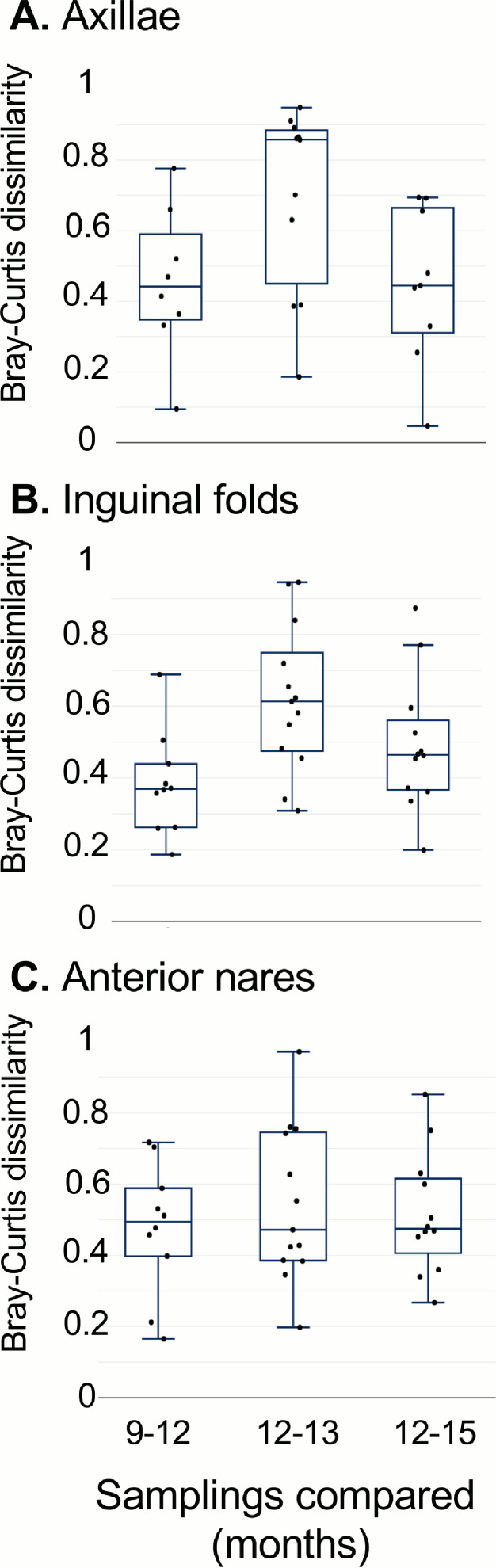
Comparison of bacterial communities over time. The Bray-Curtis dissimilarity of bacterial communities from (A) axillae, (B) inguinal folds, and (C) anterior nares samples collected from the same individual at two time points are shown in the box and whisker plots. The data are displayed with box and whisker plots, which include the median (solid horizontal line), interquartile ranges (box), and min and max (whiskers), excluding outliers. A higher value indicates a greater difference in community composition between the two samples compared. As a baseline, the samples collected at 9 and 12 months were compared. To evaluate samples collected before and after the intervention, the 12- and 13-month samples were compared. To test for a return to baseline, the samples collected at 12 months (pre-intervention) and 15 months (3 months post-intervention) were compared. The differences between intervals were compared using Wilcoxon tests. Comparing the 9–12 and 12–13 month values: axillae, *P* = 0.1; inguinal folds, *P* = 0.03; anterior nares, *P* = 1.

To determine whether the community structure was disrupted by decolonization, and whether this disruption persisted, we evaluated the change in community structure using Bray-Curtis dissimilarity, a measure of beta diversity (Fig. 5). We measured the dissimilarity between the communities from the 9- and 12-month samplings to serve as a baseline level of community stability. We then measured the dissimilarity between the communities at the 12- and 13-month samplings (i.e., before and after the decolonization intervention) to measure the effects of the treatment on community structure. Finally, we measured the dissimilarity between the 12-month and the 15-month samplings to determine whether the community had returned to its baseline state. In the inguinal folds, the difference in community structure was greater between the 12-month pre-intervention sample and the 13-month post-intervention sample than between the 9-month and 12-month samples pre-intervention (*P* = 0.03), suggesting the intervention caused significant disruption. In the axillae, there was a trend toward greater disruption, but it was not statistically significant (*P* = 0.1). No difference was observed in the nares samples (*P* = 1). The degree of change in community structure between the 9- and 12-month (both pre-intervention) samplings did not differ from the change observed between the 12-month (pre-intervention) and 15-month (3 months post-intervention) samplings for all body sites.

**FIG 6:**
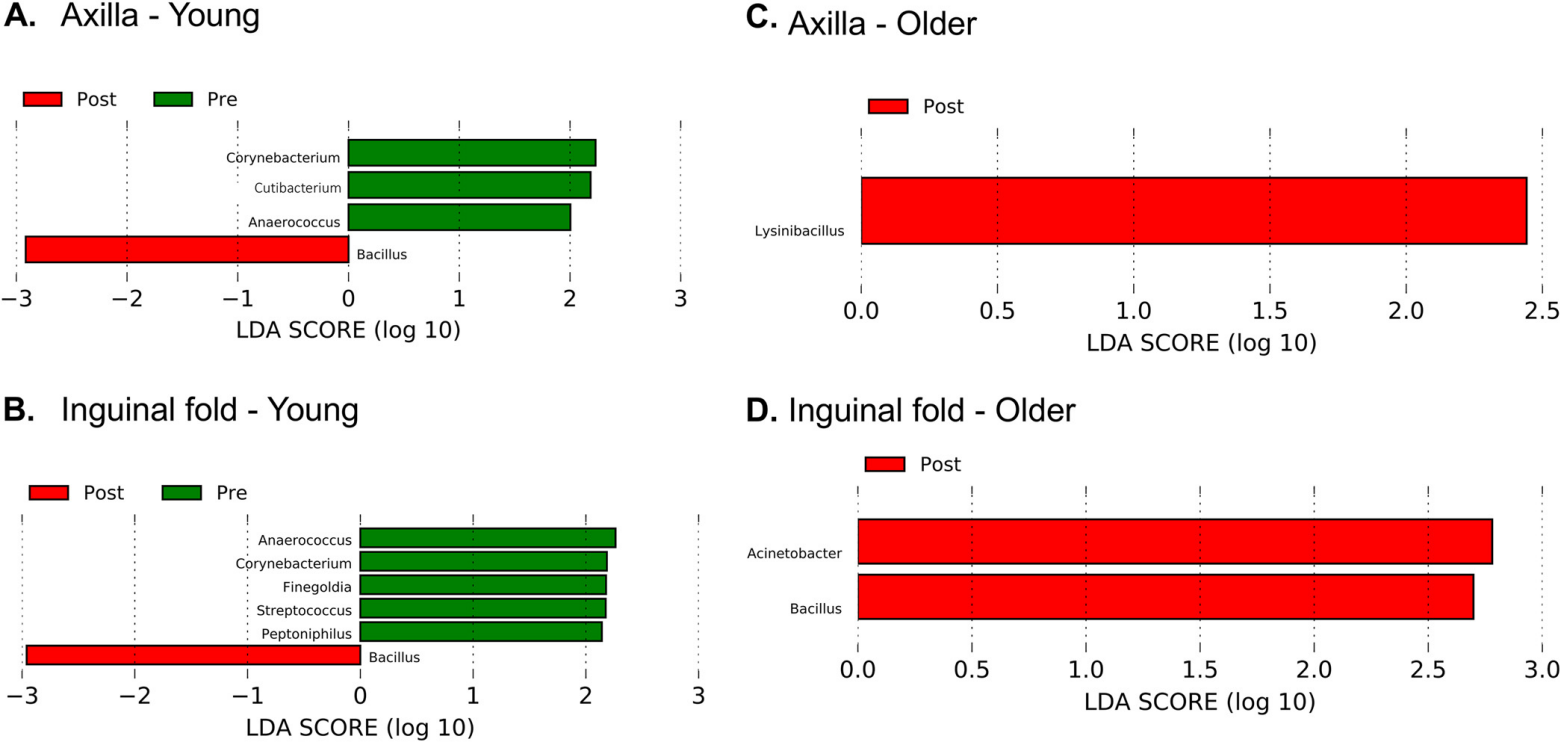
Taxa associated with pre- and post-intervention intervals. Subjects were stratified by age (young <12 years, older ≥12 years), and linear discriminant analysis (LefSe) was used to identify taxa associated with pre- and post-intervention intervals. Green bars indicate taxa associated with the pre-intervention interval, and red bars indicate taxa associated with the post-intervention interval.

**FIG 7:**
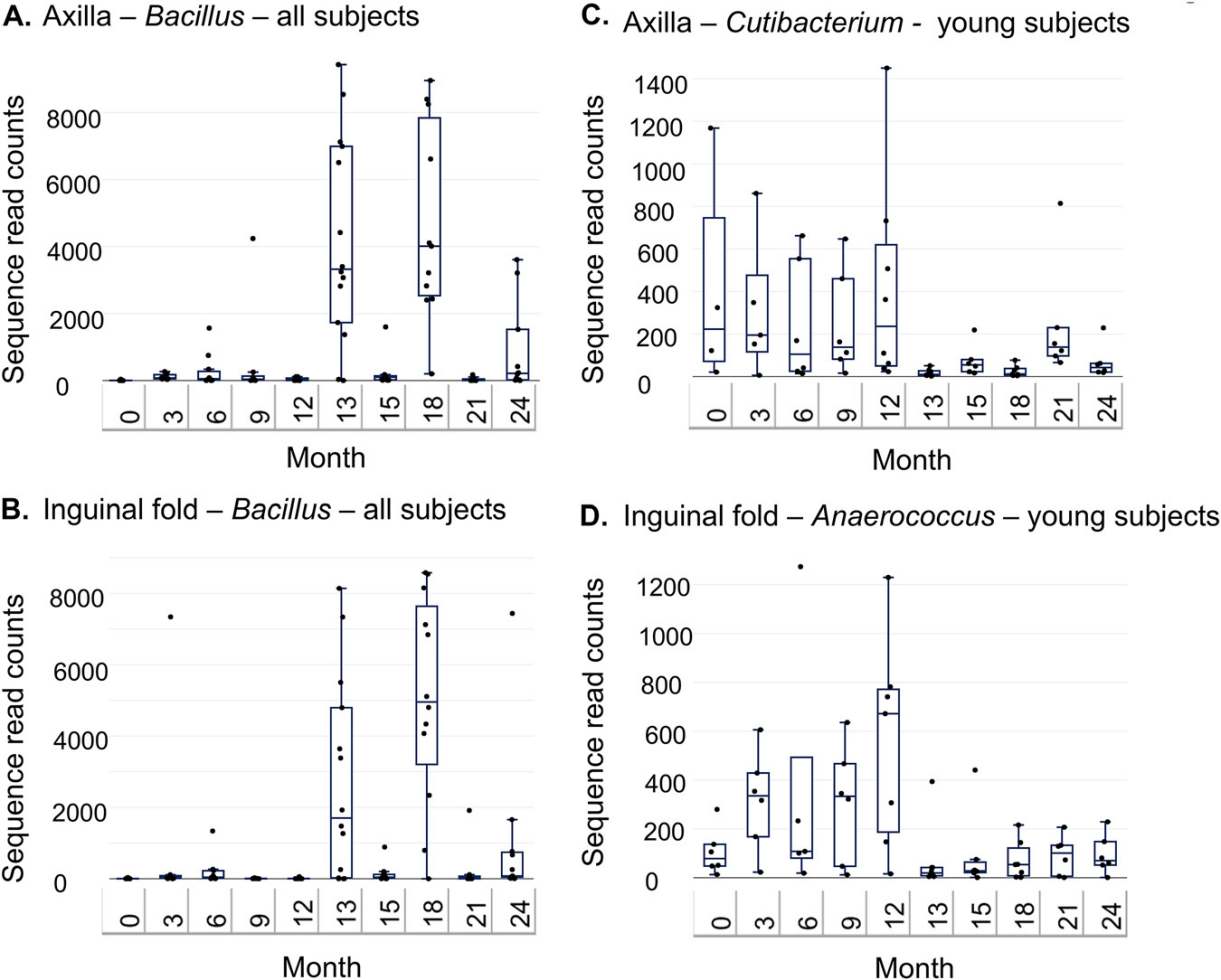
Read counts of taxa associated with pre- and post-intervention intervals. Taxa that distinguish pre- and post-intervention intervals were identified using linear discriminant analysis (LefSe). Distributions of sequence read counts (*y* axis) are shown over time (*x* axis). Read counts are from the data that were subsampled to 10,046 total reads per sample. The data are displayed with box and whisker plots, which include the median (solid horizontal line), interquartile ranges (box), and min and max (whiskers), excluding outliers.

We then evaluated whether specific taxa were affected by decolonization and whether decolonization resulted in lasting effects. We stratified by age due to the age-related differences in community composition. In the axillae and inguinal folds, we found taxa that were distinct before and after decolonization (Fig. 6). Notably, *Bacillus* was found at higher relative abundance post-decolonization in the axillae and inguinal folds from younger subjects and the inguinal folds from older subjects. The *Bacillus* levels were very low in all subjects in the observational period; post-intervention, the relative abundance increased 1 month after the prescribed intervention and continued to oscillate in alternate months (Fig. 7A and B). In younger subjects, several taxa were more predominant before decolonization (Fig. 6). For example, *Cutibacterium* and *Anaerococcus* were found at higher levels during the observational period, and they decreased and remained low throughout the post-intervention period (Fig. 7C and D). In the nares, no taxa were associated with pre- or post-intervention samples.

## DISCUSSION

The practice of decolonization with topical antimicrobials to prevent S. aureus infection has become widespread across health care and community settings (5–7). While these interventions are successful in eradicating S. aureus carriage, the collateral effects of topical antimicrobials on commensal bacterial communities is largely unexplored (4, 10, 11). Topical agents may disrupt the skin microbiota, resulting in shifts in predominant organisms and potential loss of protective microbes (22–24). Such bacterial community disruption has been associated with inflammatory skin disorders (25–28). This study demonstrates that a rigorous, prescribed decolonization regimen can lead to changes in skin bacterial communities. While some of the observed changes were transient, others persisted 1 year after the decolonization intervention.

We observed general stability in bacterial communities within each anatomic niche throughout the pre-intervention period, even in the context of sporadic bleach baths by a few household members. This is consistent with other published studies that have shown that skin bacterial communities are generally stable over time (29). However, during the post-intervention period we observed dynamic changes in diversity and sustained changes in bacterial community composition. Previous studies evaluating effects of decolonization have differed in decolonization measures prescribed, outcomes, microbial identification techniques, and sampling intervals, and thus are not directly comparable to the present study (30–32). However, in both adult and neonatal intensive care units, chlorhexidine bathing has been demonstrated to reduce microbial diversity and bacterial burden (33, 34). Furthermore, in S. aureus*-*colonized adults from community and nursing home settings, a 5-day course of mupirocin was shown to reduce S. aureus for 8 weeks after treatment (14). That study found no difference in overall alpha diversity (e.g., Shannon diversity) or in beta diversity between the pretreatment and 4-week posttreatment samples, which is consistent with our observations in the nares at that time point (Fig. 4 and 5, comparing 12- and 13-month samplings) (14). Our study contributes to the understanding of the longitudinal effects over a full year of frequently prescribed topical agents for decolonization, specifically mupirocin and bleach baths, in a household setting. The sustained disruption observed months following decolonization may be partially explained by the intensity and duration of the decolonization regimen. While this protocol is more effective at diminishing S. aureus colonization than shorter protocols (e.g., a single bleach bath), our data suggest that the increased intensity and duration of the decolonization regimen may have long-term impact on microbial ecology of the skin.

Bacterial community changes were not limited to the 4 individuals who performed the decolonization protocol. Studies have described the phenomenon of a “household microbiota” resulting from close personal contact and shared environments (35). Song and colleagues demonstrated that bacterial communities, particularly those recovered from the skin, were more similar between cohabitating individuals than those living in separate households (36). This effect was especially pronounced in larger families and households with dogs, consistent with our family comprised of 3 adults, 11 children, 5 dogs, and 3 cats. Moreover, in our household in which only 4 members underwent decolonization, we demonstrated that prescribed interventions (compared to sporadic bleach baths) can have a broader impact on other household members. This raises questions as to whether the same effects would be observed in smaller households or households in which fewer people received the intervention. The mechanisms of the shared community dynamics have yet to be determined. It is possible that bleach baths and mupirocin affect the shared microbes through direct personal contact, through household environmental reservoirs, or both. Given the broad impact of decolonization on household members, it is important to understand the implications of bacterial community disruption on susceptibility and resistance to colonization and infection with pathogenic bacteria.

A predominant concern surrounding decolonization is replacement of commensal organisms with microbes posing greater pathogenic potential. In our study, we observed increased relative abundance of Acinetobacter species in older participants after decolonization, which could lead to skin and soft tissue infections with this pathogen (37). A systematic review found that, compared to controls, patients who had used mupirocin were significantly more likely to develop infections caused by pathogens other than S. aureus (38). This could be due to loss of protective effects of commensal organisms, which protect the host from colonization and invasion by potential pathogens (13, 39–42). Our study demonstrates that the taxa affected by decolonization protocols differ in younger and older populations due to the differences in their baseline community characteristics. A study by Oh and colleagues found that children (classified as Tanner stages 1–3) had higher interpersonal variation in their bacterial communities compared with older individuals (Tanner stages 4 and 5) (25), consistent with the present study. Common respiratory pathobionts were also recovered from children, whereas more lipophilic bacteria (e.g., *Cutibacterium* and *Corynebacterium* spp.) were recovered from older individuals, likely due to the increased activity of sebaceous and apocrine glands associated with puberty. This raises the question of whether disrupting bacterial communities in children may be problematic for development of stable, healthy bacterial communities as they age. Alternatively, there might be an ideal time to disrupt a community dominated by S. aureus to allow repopulation with a more optimal set of microbes during the transition to an adult bacterial community structure.

A strength of this study is the longitudinal nature with a prescribed, intense decolonization intervention at the midpoint. This facilitates description of baseline characteristics followed by analysis of the long-term effects of the intervention in the same subjects. Moreover, while the majority of the limited studies evaluating the effects of topical antimicrobials on the skin and nasal microbiota to date have employed culture-based taxonomic identification (30–32), the 16S rRNA gene sequencing performed in the present study provides a culture-independent analysis of the bacterial communities, enabling a more comprehensive assessment. Future studies could use metagenomic shotgun sequencing rather than 16S rRNA gene sequencing, which would enable species-level taxonomic classification and facilitate analysis of the gene content of the bacterial community. Analyzing samples from members of one large household is both a strength and limitation of this study. Evaluating bacterial communities among household members controls for environmental exposures that may influence community dynamics. Furthermore, it allowed us to evaluate the effects of decolonization in both adults and children. The limited sample size of the present study, particularly when stratified by age and body site, precludes our ability to identify specific constellations of microbes associated with S. aureus colonization or development of subsequent infection.

In conclusion, as decolonization initiatives for infection prevention continue to expand, it is essential that we understand the capacity of these agents to disrupt commensal bacterial communities and associated consequences. Larger studies of more diverse patient populations are needed to understand the collateral effects of decolonization over time in people of all ages.

## MATERIALS AND METHODS

### Cohort recruitment, data, and sample collection.

To investigate CA-MRSA household transmission dynamics and prevention strategies, pediatric index patients with acute CA-MRSA skin and soft tissue infections (SSTI) were recruited as previously described (17–21). The present analysis investigated nasal and skin bacterial communities recovered from members of one enrolled household. Study personnel conducted all research visits in the participants’ home. Informed consent (and assent when appropriate) was obtained. The study design is depicted in Fig. S1 The observational period consisted of the enrollment visit and longitudinal visits 3, 6, 9, and 12 months following enrollment. At the 12-month visit, the household was randomized into a pragmatic intervention trial (20) in which the 4 household members who experienced SSTI during the observational period (i.e., the index patient and 3 household contacts) were assigned a 5-day decolonization regimen (application of 2% mupirocin to the anterior nares twice daily and soaking for ≥15 min each day in dilute bleach water [6]). Post-intervention study visits occurred 13, 15, 18, 21, and 24 months following initial study enrollment. During each study visit, participants were queried regarding the incidence of interval SSTI, antibiotic consumption, and topical antimicrobials or antiseptics applied in addition to the trial protocol. Additionally, swab samples (BD Eswab; Becton, Dickinson) were obtained following a standardized protocol from the anterior nares, axillae, and inguinal folds of each participant. S. aureus was recovered using broth-enrichment and molecular typing was performed on all recovered S. aureus isolates by repetitive-sequence PCR (repPCR) (21). The remaining swab transport medium was aliquoted and frozen at −80°C for genomic analyses. All study procedures were approved by the Washington University Institutional Review Board.

### 16S rRNA gene sequencing and analysis.

The V1–V2 region of the 16S rRNA gene was amplified and sequenced on the MiSeq platform (Illumina, San Diego, CA, USA). Sequences were processed and taxonomically classified using Mothur (43, 44). Data were subsampled to 10,046 reads per sample for comparisons. Thirty-seven of the 363 samples (10%) had read counts <10,000 and were removed from further analysis. See Supplemental Methods for details. Sequence data have been deposited in the Sequence Read Archive under Bioproject PRJNA788575.

### Statistical analyses.

The R statistical program was used for statistical analysis and plotting (45). The R libraries vegan (46), labdsv (47), plotly (48), and lme4 (49) were used to calculate diversity, create NMDS plots, and explore longitudinal trends. Differential representation of specific taxa was evaluated using LEfSe (50). For some analyses we stratified the subjects into two groups based on age (<12 and ≥12 years old at the start of the study), as significant changes occur in the skin microbiome coincident with sexual maturation (25). See Supplemental Methods for details.
