# Supplementary material for: Longitudinal Dynamics of Skin Bacterial Communities in the Context of Staphylococcus aureus Decolonization
Source: Microbiol Spectr. 2022 Apr 6;10(2):e02672-21. doi: 10.1128/spectrum.02672-21 (PMC9045213; doi:10.1128/spectrum.02672-21)
Supplement: SUPPLEMENTAL FILE 1 — Supplemental material. Download SPECTRUM02672-21_Supp_1_seq2.pdf, PDF file, 0.2 MB [file spectrum02672-21_supp_1_seq2.pdf]

Supplemental Methods

16S rRNA gene sequencing

DNA was extracted using the QIAamp BiOstic Bacteremia DNA Kit (Qiagen, Germantown, MD, USA). Negative controls (blank eluate passed through the DNA extraction process) were included to assess background levels of contamination. The V1–V2 region of the 16S rRNA gene was amplified and Illumina adapter sequences added using the following primers:

Forward: AATGATACGGCGACCACCGAGATCTACAC[8-bp-index]TCGTCGGCAGCGTCAGATGTGTATAAGAGACAG[6-bp-pad]AGAGTTTGATCMTGGCTCAG

Reverse: CAAGCAGAAGACGGCATACGAGAT[8-bp-index]GTCTCGTGGGCTCGGAGATGTGTATAAGAGACAG[6-bp-pad]CTGCTGCCTYCCGTA.

Dual-indexed sequencing libraries were pooled in equimolar concentrations. Negative controls were added to the pool in their entirety. Amplicons were sequenced on the Illumina MiSeq platform using the 2x300 V2 Reagent Kit (Illumina, San Diego, CA, USA).

Sequence analysis

Sequences were processed using Mothur [1, 2]. UCHIME was used to screen chimeric sequences [3]. Sequences were taxonomically classified using a naïve Bayesian classifier with the Ribosomal Database Project training set version 9 [4]. Taxonomy was assigned to the lowest taxonomic level at which 80% confidence was met. Data were subsampled to 10,046 reads per sample for comparisons. 37 of the 363 samples (10%) had read counts <10,000 and were removed from further analysis.

Statistical analyses

The R statistical program was used for statistical analysis and plotting [5]. Alpha diversity (richness and Shannon diversity) and beta diversity (Bray-Curtis dissimilarity) measures were

calculated with the R library vegan [6]. Non-metric multidimensional scaling (NMDS) was evaluated with the R package labdsv [7], and Adonis was used to test for differences between groups using the R library vegan [6]. Box plots and bar charts were generated with the R library plotly [8]. Linear mixed-effects models were generated with the R library lme4 to explore longitudinal trends [9]. In these models, the response variable was Shannon diversity; fixed variables were age and sampling visit; subjects were included as random effects. P-values were calculated using a likelihood ratio test comparing models with and without the effect in question. Differential representation of specific taxa was evaluated using LEfSe [10]. For LEfSe, the alpha values for the factorial Kruskal-Wallis test among classes and the pairwise Wilcoxon tests between subclasses were 0.05. The threshold on the logarithmic linear discriminate analysis (LDA) score was 2.0 for discriminative features. For some analyses we stratified the subjects into two groups based on age (<12 and $\geq$12 years old at the start of the study), as significant changes occur in the skin microbiome coincident with sexual maturation [11].

1. Kozich JJ, Westcott SL, Baxter NT, Highlander SK, Schloss PD. Development of a dual-index sequencing strategy and curation pipeline for analyzing amplicon sequence data on the MiSeq Illumina sequencing platform. Appl Environ Microbiol **2013**; 79(17): 5112-20.
2. Schloss PD, Westcott SL, Ryabin T, et al. Introducing mothur: open-source, platform-independent, community-supported software for describing and comparing microbial communities. Appl Environ Microbiol **2009**; 75(23): 7537-41.
3. Edgar RC, Haas BJ, Clemente JC, Quince C, Knight R. UCHIME improves sensitivity and speed of chimera detection. Bioinformatics **2011**; 27(16): 2194-200.
4. Wang Q, Garrity GM, Tiedje JM, Cole JR. Naive Bayesian classifier for rapid assignment of rRNA sequences into the new bacterial taxonomy. Appl Environ Microbiol **2007**; 73(16): 5261-7.
5. Team RC. R: A language and environment for statistical computing. In: R Foundation for Statistical Computing V, Austria, **2016**.
6. Oksanen J, Blanchet FG, Friendly M, et al. vegan: Community Ecology Package. R package version 2.4-3. Available at: https://CRAN.R-project.org/package=vegan.
7. Oksanen J, Blanchet FG, Friendly M, et al. labdsv: Ordination and Multivariate Analysis for Ecology. R package version 1.8-0. Available at: https://cran.r-project.org/package=labdsv.
8. Sievert C, Parmer C, Hocking T, et al. plotly: Create Interactive Web Graphics via 'plotly.js'. R package. version 4.7.1. Available at: https://CRAN.R-project.org/package=plotly.
9. Bates D, Machler M, Bolker B, Walker S. Fitting linear mixed-effects models using lme4. Journal of Statistical Software **2015**; 67(1): 1-48.

10. Segata N, Izard J, Waldron L, et al. Metagenomic biomarker discovery and explanation. Genome Biol **2011**; 12(6): R60.
11. Oh J, Conlan S, Polley EC, Segre JA, Kong HH. Shifts in human skin and nares microbiota of healthy children and adults. Genome Med **2012**; 4(10): 77.

Supplemental Figure

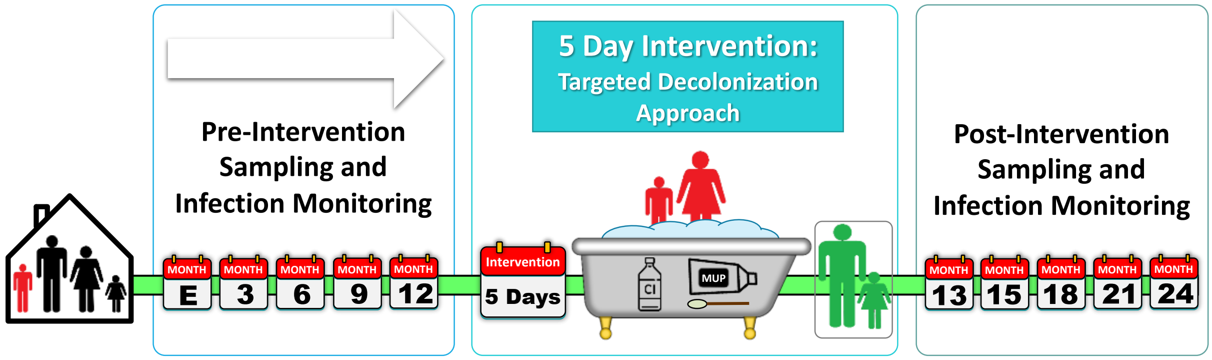

Supplemental Figure 1. Longitudinal Study Design. The observational period consisted of the enrollment visit and longitudinal visits 3, 6, 9, and 12 months following enrollment. At the 12-month visit, the household was randomized into a pragmatic intervention trial in which the 4 household members who experienced SSTI during the observational period (i.e., the index patient and 3 household contacts) were assigned a 5-day decolonization regimen (application of 2% mupirocin to the anterior nares twice daily and soaking for ≥15 minutes each day in dilute bleach water). Post-intervention study visits occurred 13, 15, 18, 21, and 24 months following initial study enrollment. At each study visits, swab samples were collected from the anterior nares, axilla, and inguinal fold for each participant. Participants were also queried regarding the incidence of interval SSTI, antibiotic consumption, and topical antimicrobials or antiseptics applied in addition to the trial protocol.
